# MRSA USA300 at Alaska Native Medical Center, Anchorage, Alaska, USA, 2000–2006

**DOI:** 10.3201/eid1801.110746

**Published:** 2012-01

**Authors:** Michael Z. David, Karen M. Rudolph, Thomas W. Hennessy, Diana L. Zychowski, Karthik Asthi, Susan Boyle-Vavra, Robert S. Daum

**Affiliations:** The University of Chicago, Chicago, Illinois, USA (M.Z. David, D.L. Zychowski, K. Asthi, S. Boyle-Vavra, R.S. Daum);; Centers for Disease Control and Prevention, Anchorage, Alaska, USA (K.M. Rudolph, T.W. Hennessy)

**Keywords:** MRSA, Staphylococcus aureus, methicillin-resistant S. aureus, staphylococci, skin and soft tissue infections, Alaska, epidemiology, genotyping, multilocus sequence typing, bacteria, antibiotic resistance, bacteria, community-associated infections

## Abstract

To determine whether methicillin-resistant *Staphylococcus aureus* (MRSA) USA300 commonly caused infections among Alaska Natives, we examined clinical MRSA isolates from the Alaska Native Medical Center, Anchorage, during 2000–2006. Among Anchorage-region residents, USA300 was a minor constituent among MRSA isolates in 2000–2003 (11/68, 16%); by 2006, USA300 was the exclusive genotype identified (10/10).

Methicillin-resistant *Staphylococcus aureus* (MRSA) isolates, once concentrated among patients who had contact with the health care environment, have become epidemic among otherwise healthy populations in the United States. In the 48 contiguous states, community-associated MRSA skin and soft tissue infections (SSTIs) are predominantly caused by strain USA300 (*1*). In contrast, in 1996, 2000, and 2004–2006, in rural southwestern Alaska, we found that USA300 was rarely isolated, although community-associated MRSA SSTIs were common. Instead, sequence type (ST) 1, the type of USA400 isolates, was more common (*2*), as others have found in northern Canada (*3*). We wondered whether, over time, USA300 might replace USA400 among Alaska Natives as it has elsewhere in North America (*4**,**5*).

To investigate this possibility, we conducted surveillance at the Alaska Native Medical Center (ANMC). ANMC is the primary hospital for Alaska Natives residing in the Anchorage area and the statewide referral hospital for the Alaska Tribal Health System.

## The Study

During 2000–2003, 695 clinical MRSA isolates were obtained by the Clinical Microbiology Laboratory of ANMC. A convenience sample of 567 isolates was collected and termed the retrospective collection. This collection was stratified by year of isolation and by 3 geographic regions of Alaska in which patients resided: 1) the Anchorage region (Anchorage, the Mat-Su region, and the Aleutian Islands); 2) the region of southwestern Alaska previously studied (*2*); and 3) all other regions. A randomly selected sample of 163 (28.7%) of the 567 isolates, stratified by year of isolation, was chosen for genotyping, including 20% of the isolates from Anchorage-region residents, 20% from residents of southwestern Alaska, and all isolates from residents of other regions (Table 1).

**Table 1:** Overview of patient characteristics from retrospective (2000–2003) and prospective (2004–2006) collections from Alaska Native Medical Center, Anchorage, Alaska, USA, by location of patient residence*

Characteristic	No. (%) samples							
	Anchorage region			Southwestern Alaska region			Other Alaska regions	
	Retrospective collection, n = 68	Prospective collection, n = 29		Retrospective collection, n = 33	Prospective collection, n = 23		Retrospective collection, n = 62	Prospective collection, n = 9
Sex								
M	34 (50)	10 (34)		22 (67)	12 (52)		41 (66)	7 (78)
F	34 (50)	19 (66)		11 (33)	11 (48)		21 (34)	2 (22)
Age group, y								
0–2	1 (2)	3 (10)		4 (12)	1 (4)		4 (7)	0
3–12	7 (10)	2 (7)		4 (12)	6 (26)		4 (7)	0
13–20	9 (13)	2 (7)		3 (9)	1 (4)		3 (5)	1 (11)
21–39	24 (35)	10 (35)		11 (33)	7 (30)		7 (11)	0
40–59	24 (35)	11 (38)		5 (15)	4 (17)		24 (39)	4 (44)
>60	3 (4)	1 (4)		6 (18)	4 (17)		20 (32)	4 (44)
Clinical specimen								
Blood	1 (2)	1 (4)		0	1 (4)		0	0
Bone or joint	1 (2)	0		0	1 (4)		1 (2)	1 (11)
Respiratory tract	3 (4)	1 (4)		6 (18)	2 (9)		22 (36)	2 (22)
Skin or soft tissue	56 (82)	25 (86)		26 (79)	15 (65)		33 (53)	3 (33)
Urine	1 (2)	1 (4)		0	0		1 (2)	0
Other†	6 (9)	1 (4)		1 (3)	4 (17)		4 (7)	3 (33)
Unknown	0	0		0	0		1 (2)	0
Site of care (prospective only)								
Inpatient	NA	2 (7)		NA	11 (48)		NA	5 (56)
Outpatient	NA	27 (93)		NA	12 (52)		NA	4 (44)
Emergency	NA	0		NA	0		NA	0

*NA, not available. †Liver abscess, pleural fluid, tracheal culture after tracheostomy placement, surgical drain fluid culture, eye culture from eye with conjunctivitis, cultures from other eye specimens, culture from soft tissue of the neck, culture from ear with otitis media, and cultures from other ear specimens.

The prospective collection collected in 2004–2006 consisted of the first 5 clinical MRSA isolates obtained each month by the ANMC Clinical Microbiology Laboratory from different patients. Although 177 MRSA isolates had been collected, 2 were not available, and 2 lacked the *mecA* gene by PCR, leaving 173 isolates for further study. Genotyping was carried out on a random sample of 20% of isolates from this collection, stratified by year of isolation from the Anchorage-region patients, and on samples from all patients from all other regions (Table 1).

Clinical and demographic information was collected about the patients comprising the retrospective and prospective isolate groups. Site of care was recorded only for the prospective collection. Active surveillance for MRSA was not performed at ANMC during 2000–2006.

Isolates were genotyped by multilocus sequence typing (MLST), and clonal complexes (CCs) were assigned to closely related sequence types as described (*6**,**7*). Staphylococcal cassette chromosome *mec* (SCC*mec*) typing was performed (*8*), and the presence of Panton-Valentine leukocidin (PVL) genetic determinants was assessed as described (*9*). Additionally, to clarify the relationship between ST and typing by pulsed-field gel electrophoresis (PFGE), a random sample of strains that were ST8 and ST1 were tested by PFGE as described (*10*). Control strains were USA300-LAC for USA300 and strain 649, a clinical strain identical to MW2 by PFGE, for USA400. Antimicrobial drug susceptibilities were determined by using automated testing (bioMérieux Vitek, Durham, NC, USA). The D-zone test for inducible clindamycin resistance was performed for isolates resistant to erythromycin and susceptible to clindamycin by single-agent testing (*11*). Results were compared by χ^2^ or Fisher exact tests using Stata version 11 (StataCorp LP, College Station, TX, USA).

The patients in the 20% Anchorage-region retrospective sample (n = 68), in the 20% retrospective sample from the region of southwestern Alaska (n = 33), and in the 20% Anchorage-region prospective sample (n = 29) did not differ significantly by demographic characteristics from the larger sampled groups (data not shown). Isolates in the combined retrospective and prospective collections were distributed among 14 MLST types (Table 2). Nearly all CC1 (99%), CC8 (98%), and CC30 (100%) isolates were positive for Panton-Valentine leukocidin (PVL); all isolates in these 3 CCs carried SCC*mec* IV. No CC5 or CC45 isolates were PVL positive. Among CC5 isolates, 41/44 (93%) carried SCC*mec* II. All CC45 isolates carried SCC*mec* IV.

**Table 2:** MRSA isolate characteristics, combined retrospective and prospective collection samples by region of patient residence, Alaska, USA, 2000–2006*†

CC and ST	No. (%) patients		
	Anchorage region, n = 97	Southwestern region, n = 56	Other regions, n = 71
CC1‡			
ST1	25 (26)	43 (77)	10 (14)
CC5			
ST5	11 (11)	2 (4)	16 (23)
ST105	1 (1)	2 (4)	9 (13)
ST225	0	0	1 (1)
ST231	0	1 (2)	1 (1)
CC8§			
ST8	32 (33)	2 (4)	9 (13)
CC30			
ST30	14 (14)	3 (5)	16 (23)
ST30slv	3 (3)	2 (4)	0
CC45			
ST45	1 (1)	0	0
ST45slv	1 (1)	0	0
ST54	1 (1)	0	0
CC59			
ST59	7 (7)	1 (2)	7 (10)
ST969	0	0	2 (3)
ST969slv	1 (1)	0	0
SCC*mec* type			
II	10 (10)	4 (7)	29 (41)
IV	88 (91)	52 (93)	39 (55)
c-2.5	1 (1)	0	3 (4)

*MRSA, methicillin-resistant *Staphyloccus aureus*; CC, clonal complex; ST, sequence type; slv, single locus variant; SCC*mec*, staphylococcal cassette chromosome *mec;* PFGE, pulsed-field gel electrophoresis. †Among 61 isolates genotyped from the prospective cohort, 18 were from inpatients (ST1 [n = 9], ST105 [n = 1], ST231 [n = 1], ST5 [n = 3], and ST8 [n = 4]), and 43 were from outpatients (ST1 [n = 14], ST105 [n = 1], ST30 [n = 2], ST30slv [n = 1], ST5 [n = 3], and ST8 [n = 22]). ‡CC1 has recently been changed to CC15 by the administrators of the multilocus sequence typing system. A randomly selected sample of 14 ST1 isolates that carried the SCC*mec* type IV element and were Panton-Valentine leukocidin positive underwent PFGE, and 93% (13/14) were USA400. §42/43 ST8 strains identified in the study carried the SCC*mec* type IV element and were Panton-Valentine leukocidin positive. They are all considered to be USA300 because 30 of the 42 were randomly selected to undergo PFGE, and 100% (30/30) had the USA300 pulsotype.

PFGE was performed on 30 ST8 and 14 ST1 isolates that were PVL positive and contained SCC*mec* IV. Of the ST8 isolates, 100% (30/30) were USA300, and of the ST1 isolates, 93% (13/14) were USA400.

When our sample was adjusted to account for the sampling strategy, we were able to estimate the genotypic spectrum of all isolates. In the retrospective collection, an estimated 12% (67/567) of MRSA isolates were ST8, and 42% (236/567) were ST1. Similarly, we estimated in the prospective collection that 61% (105/173) were ST8, and 25% (44/173) were ST1.

ST8 isolates were first identified among Anchorage-region patients in 2002 and accounted for 31% (5/16) of the genotyped isolates in that year; this increased to 100% (10/10) in 2006. When isolates from 2000–2003 were compared with those from 2004–2006, the proportion of ST8 isolates obtained from Anchorage-region residents increased significantly (11/68, 16% vs. 21/29, 72%; p<0.001); among patients from southwestern Alaska (p = 0.2) and from other regions (p = 0.8), the percentage of ST8 isolates also increased, but the increase was not significant (Figure).

**Figure:**
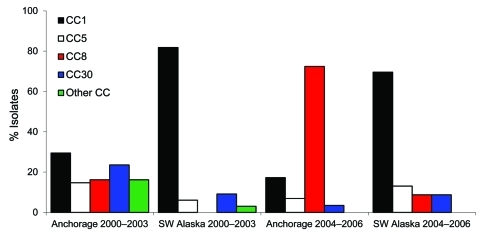
Percentage of clonal complex (CC) 1, CC5, CC8, CC30, and other CC methicillin-resistant *Staphylococcus aureus* isolates from residents of the Anchorage region and the region of southwestern Alaska, USA, 2000–2003 and 2004–2006.

MRSA isolates from Anchorage-region patients were obtained more often from an SSTI (81/97, 84%) than those from other regions (77/127, 61%; p<0.001). In the prospective collection, isolates from the Anchorage region were more likely to be from outpatients (27/29, 93%) than were those from other regions (17/33, 52%) (p<0.001), and outpatients were more likely to have an SSTI (39/43, 91%) than were inpatients (5/18, 28%; p<0.001). In 2006, when all MRSA isolates obtained from Anchorage-region residents were ST8, 9/10 were from outpatients with SSTIs.

Among the Anchorage-region patients, SSTIs accounted for only 33% (4/12) of CC5 isolates, compared with 96% (24/25) of CC1, 91% (29/32) of CC8, 100% (17/17) of CC30, and 100% (3/3) of CC45 isolates. Also among these patients, CC8 (94%), CC30 (100%), and CC59 (100%) isolates were almost all susceptible to clindamycin. Of all CC1 isolates, 16/25 (64%) were resistant to clindamycin; 14/16 (88%) were not susceptible by virtue of a positive D-zone test result.

## Conclusions

We documented the emergence and rapid dominance of USA300 among clinical MRSA isolates from Anchorage-region patients who received treatment at the ANMC in 2002–2006. This complete strain replacement by 2006 suggests that Anchorage-region patients were exposed to a growing reservoir of USA300 during this era. In contrast, in 2006, USA300 still remained a less common cause of MRSA infections among ANMC patients drawn from other regions of Alaska, perhaps because patients from other regions were less likely to have been referred to ANMC for uncomplicated SSTIs.

Of the tested PVL-positive ST8 MRSA isolates bearing SCC*mec* IV, we confirmed that 100% (30/30) were USA300 by PFGE. This finding has useful implications for comparing MLST and PFGE typing methods.

ST1 isolates frequently had inducible clindamycin resistance, whereas strains with PVL and SCC*mec* IV of other genetic backgrounds rarely did. This may explain the seemingly anomalous data from the USA400 era in Chicago that 31/33 (94%) of clindamycin-susceptible, erythromycin-resistant strains of MRSA were D-zone test positive (*12*).

Documented introduction of USA300 has not resulted in strain replacement in Europe (*13*), Asia (*14*), or Australia (*15*). That USA300 emerged and came to predominate among Anchorage-region residents, mirroring the process that occurred earlier in several cities in North America (*4**,**5*), suggests that some characteristic of USA300 provides a survival advantage, enhanced virulence, or both, relative to other MRSA pulsotypes in those regions. The rapid appearance and emergence of USA300 at ANMC in 2002–2006 were remarkable, but its limited global spread remains unexplained.
